# Supplementary material for: Sustained-input switches for transcription factors and microRNAs are central building blocks of eukaryotic gene circuits
Source: Genome Biol. 2013 Aug 23;14(8):R85. doi: 10.1186/gb-2013-14-8-r85 (PMC4054853; doi:10.1186/gb-2013-14-8-r85)
Supplement: Additional file 5 — HTML Browsable Motif Output. Zipped folder containing all WaRSwap and FANMOD motif output, viewable in a web browser. [file gb-2013-14-8-r85-S5.ZIP › HTML_browsable_motif_output/FANMOD_ath_tair9/sigs_fanmodm-2000.pvals.heatmaps.html/motif_id_46_000101110_tftype_ath_upstream_-1000_0.html]

```
BG_MODEL = FANMOD
MOTIF_ID = 46_000101110
TF_TYPE = ath
UPSTREAM = -1000_0


PVals
FN_0.2	FN_0.4	FN_0.6	FN_0.8
dg_60.genes	0.059	0	0.869	0.4
dg_70.genes	0.057	0	0.882	0.383
dg_80.genes	0.06	0	0.868	0.414

ZScores
FN_0.2	FN_0.4	FN_0.6	FN_0.8
dg_60.genes	1.529	3.977	-1.243	-0.704
dg_70.genes	1.527	4.124	-1.243	-0.685
dg_80.genes	1.542	4.19	-1.201	-0.728

StDevs
FN_0.2	FN_0.4	FN_0.6	FN_0.8
dg_60.genes	35.297	16.593	5.642	0.839
dg_70.genes	35.552	16.069	5.61	0.83
dg_80.genes	34.533	15.799	5.741	0.847
```
